# Metabolic Response to an Individualized Multimodal Treatment Strategy in Advanced Pancreatic Adenocarcinoma: A Case Report

**DOI:** 10.3390/curroncol33080489

**Published:** 2026-08-19

**Authors:** Mehmet Salih İyikesici, Oral Oncul, Metin Hallaç, Sena Şen, Şirin Taflan, Selin Oncul, Tomas Duraj, Thomas N. Seyfried

**Affiliations:** 1 Neolife Oncology Centre, 34340 Istanbul, Türkiye; drsalihiyikesici@gmail.com (M.S.İ.); drmetinhalac@gmail.com (M.H.); sirin_budak@hotmail.com (Ş.T.); 2 Department of Infectious Diseases and Clinical Microbiology, Division of Internal Medicine, Istanbul Faculty of Medicine, Istanbul University, 34093 Istanbul, Türkiye; 3 Department of Basic Oncology, Oncology Institute, Istanbul University, 34093 Istanbul, Türkiye; sena.sen@istanbul.edu.tr; 4 Medical School, Kings College of London, London WC2R 2LS, UK; selin.oncul@kcl.ac.uk; 5 Department of Biology, Boston College, Boston, MA 02467, USA; tomas.duraj@bc.edu (T.D.); thomas.seyfried@bc.edu (T.N.S.)

**Keywords:** pancreatic adenocarcinoma, ^18^F-FDG PET/CT, metabolic response, combined multimodal therapy, low-dose/metronomic chemotherapy, case report

## Abstract

**Background**: Metastatic pancreatic adenocarcinoma carries a dismal prognosis, and treatment options after progression on standard chemotherapy remain limited. We report a metabolic response in a patient with drug-resistant disease treated with a combined multimodal regimen that paired dose-reduced (“activated”) multi-agent chemotherapy with targeted agents and metabolic/microenvironmental support, set against an unusually long overall survival. **Case Presentation**: A 46-year-old man was diagnosed with inoperable, locally advanced/stage IV pancreatic adenocarcinoma in June 2023 and maintained disease control for approximately 2.5 years on a combined multimodal regimen, termed here metabolically controlled oncologic therapy (MCOT): dose-reduced chemotherapy given with regional hyperthermia, hyperbaric oxygen, and a carbohydrate-restricted diet. In February 2026 he developed local recurrence and diffuse liver metastases, and by May 2026 had deteriorated rapidly, with ^18^F-FDG PET/CT showing a peak SUVmax of 18.2 and CA 19-9 of 2788 U/mL. His regimen was intensified to a low-dose, multi-agent “activated” chemotherapy protocol combined with lenvatinib, everolimus, hyperthermia, and hyperbaric oxygen. **Clinical Findings and Outcomes**: One month later, his performance status had recovered from ECOG PS 2 to PS 0, CA 19-9 was 31 U/mL, and follow-up PET/CT showed a 63.7% decrease in SUVmax (from 18.2 to 6.6). Treatment-related adverse events included Grade 1 nausea, Grade 1 vomiting, and thrombocytopenia; no Grade 2 or higher non-hematological toxicity was observed. **Conclusions**: In selected patients who are considered to have exhausted standard options, a combined multimodal regimen pairing dose-reduced chemotherapy with metabolic and microenvironmental support may produce a well-tolerated metabolic response. As a single, uncontrolled observation, these findings cannot establish causality and require confirmation in prospective, controlled studies.

## Simple Summary

Advanced pancreatic cancer is one of the most difficult cancers to treat, and patients whose disease progresses after standard chemotherapy have very limited options. This case describes a patient with advanced pancreatic cancer who achieved long-term disease control using a treatment approach that combined reduced-dose chemotherapy with supportive metabolic therapies. After the cancer recurred and spread to the liver, an intensified version of the same strategy led to improvement in symptoms, physical condition, and tumor activity on imaging within one month, while causing only mild side effects. Although a single case cannot prove that this treatment is effective, it suggests that combining low-dose chemotherapy with approaches targeting the tumor environment and metabolism may warrant further investigation.

## 1. Introduction

Pancreatic ductal adenocarcinoma is among the deadliest solid tumors, reflecting its late presentation, early metastatic spread, and limited responsiveness to systemic therapy; an estimated 510,000 new cases and 467,000 deaths occurred worldwide in 2022, and five-year survival of roughly 12% remains the lowest of any major cancer [1]. First-line FOLFIRINOX extends median overall survival to 11.1 months in metastatic disease, against 6.8 months for gemcitabine alone [2], while gemcitabine plus nab-paclitaxel achieves a median of about 8.5 months [3]; options after progression remain few and modest in effect. Recently, daraxonrasib resulted in promising survival benefit in patients with RAS G12 mutations, but response to therapy was not universal and resistance mechanisms are likely to develop [4,5]. Because pancreatic cancer is typically asymptomatic in its early stages, most patients are diagnosed only after the disease has become locally advanced or metastatic, precluding potentially curative treatment. Consequently, improving early detection through more sensitive biomarkers and diagnostic strategies has become a major research priority, with the potential to substantially improve patient outcomes [6]. The absence of effective screening methods for the general population remains one of the major barriers to early diagnosis.

In patients who develop multidrug resistance, there is growing interest in pairing chemotherapy with adjunctive measures aimed at tumor metabolism and the microenvironment. The rationale begins with the reliance of cancer cells on aerobic glycolysis even in the presence of oxygen, the Warburg effect [7]; in preclinical models, a ketogenic diet alone did not slow pancreatic tumor growth, but combined with chemotherapy it roughly tripled median survival [8]. Hyperthermia can raise tumor blood flow and chemosensitivity, and hyperbaric oxygen can ease tumor hypoxia and associated drug resistance [9]; Seyfried et al. proposed combining such chronic metabolic stress with acute oxidative stressors as a low-toxicity strategy [10,11]. Clinical evidence for these approaches in pancreatic cancer nonetheless remains limited, drawn largely from small series.

In patients with metastatic pancreatic ductal adenocarcinoma who experience disease progression despite multiple lines of systemic therapy, therapeutic options become increasingly limited. In the present case, the patient had progressive, treatment-resistant disease after multiple lines of chemotherapy, ultimately developing local progression and diffuse liver metastases. Given the limited remaining standard therapeutic options and the patient’s declining clinical status, an individualized, non-standard treatment approach was considered. The intensified regimen combined dose-reduced, frequently administered chemotherapy with metabolic conditioning, regional hyperthermia, hyperbaric oxygen, and targeted therapy, aiming to address tumor-cell metabolism, hypoxia, drug delivery, and potential mechanisms of treatment resistance simultaneously, while limiting the toxicity associated with full-dose multidrug chemotherapy.

We describe a patient with advanced, refractory pancreatic adenocarcinoma in whom this combined approach, termed here metabolically controlled oncologic therapy (MCOT), first delivered approximately 2.5 years of survival, and in whom a subsequent recurrence, treated with an intensified, low-dose “activated” version of the same regimen, was followed within one month by clinical improvement and a metabolic response on ^18^F-FDG PET/CT. The report follows the CARE 2023 guideline.

## 2. Case Report

### 2.1. Subsection

A 46-year-old man (height 170 cm, weight 95 kg), with no relevant family or psychosocial history, presented with abdominal pain, anorexia, nausea, and vomiting, and on 12 June 2023 was diagnosed with inoperable, locally advanced/stage IV pancreatic adenocarcinoma (Table 1). Histopathological examination of an endoscopic ultrasound-guided fine-needle aspiration biopsy obtained from a pancreatic body mass measuring approximately 4 cm and invading adjacent vascular structures confirmed pancreatic ductal adenocarcinoma (PDAC). In addition to malignant ductal epithelial cells, the specimen contained numerous non-neoplastic ductal epithelial cells within a background of chronic pancreatitis. Baseline 18F-FDG PET/CT showed increased pancreatic metabolic activity (SUVmax 2.4), CA 19-9 was 6.2 U/mL, and performance status was ECOG PS 0. No radiotherapy or other local ablative treatment was administered during the course of the disease.

### 2.2. First Treatment Phase and Long-Term Control (2023–2026)

The patient received an integrated, combined multimodal program—referred to as “metabolically controlled oncologic therapy” (MCOT) for consistency with prior reports, but more accurately described as multimodal therapy combining dose-reduced chemotherapy with metabolic and microenvironmental measures. Standard chemotherapy doses were reduced by 40–70% and administered every 15 days for three months, along with regional hyperthermia, hyperbaric oxygen therapy, a dietitian-supervised low-carbohydrate diet with complete restriction of dietary carbohydrates, and supportive treatments intended to suppress the tumor microenvironment. After this three-month intensive phase, treatment continued as maintenance, given monthly or every other month.

Systemic chemotherapy consisted of gemcitabine–oxaliplatin (GEMOX) and FOLFIRINOX-based low-dose (40–70%) regimens. The program achieved durable disease control, and the patient remained clinically stable for three years, until February 2026. Since median overall survival in metastatic pancreatic adenocarcinoma is generally under a year, this represents disease control well beyond what would ordinarily be expected.

### 2.3. Local Progression, Progression, and Metabolic Activation (February–May 2026)

**Figure 1:**
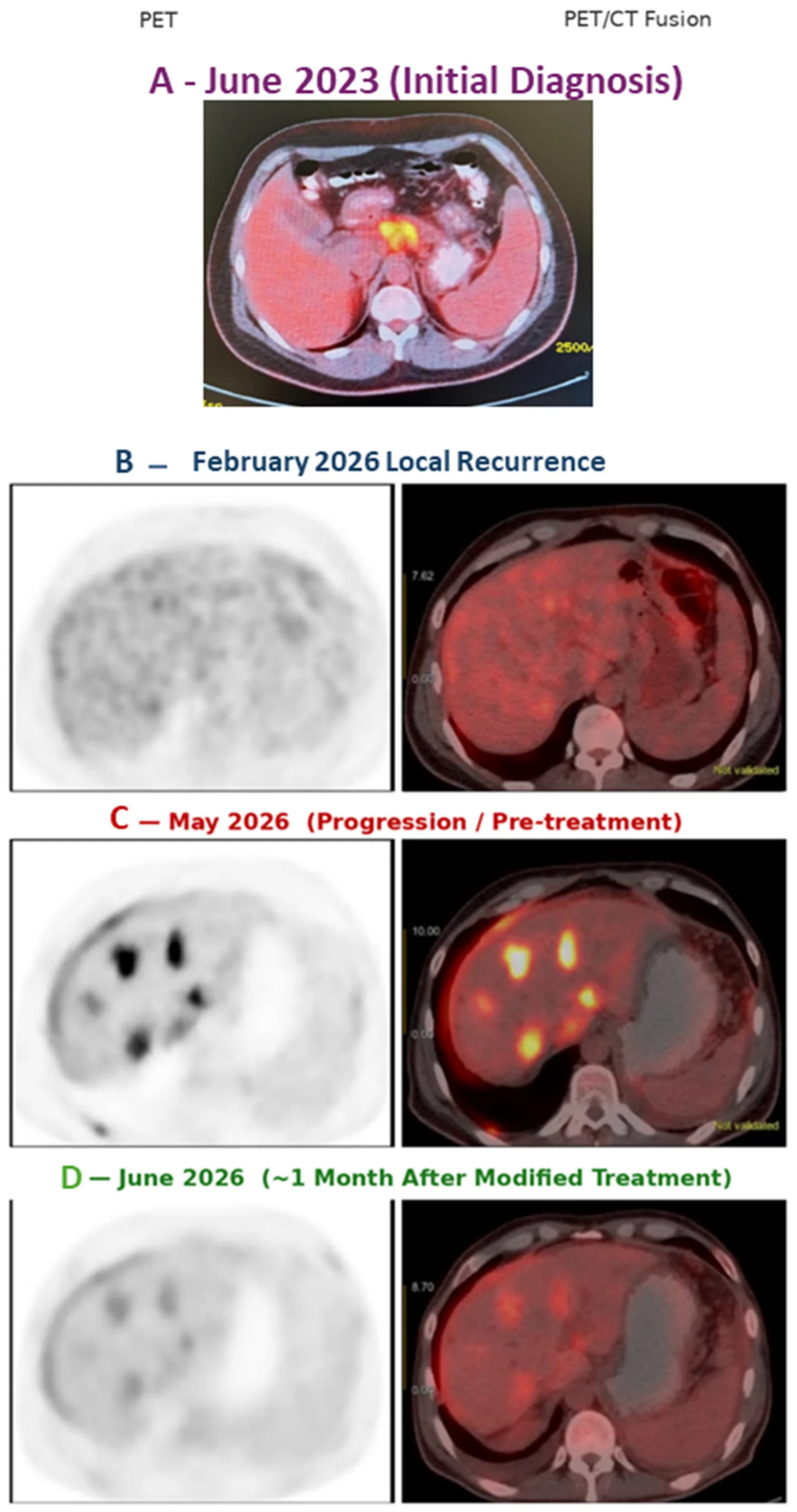
Serial 18F-FDG PET/CT imaging during the patient’s clinical course. (**A**): baseline PET/CT at diagnosis (June 2023) demonstrating FDG uptake in the primary pancreatic lesion (SUVmax 2.4). (**B**): PET/CT performed at local recurrence (February 2026). (**C**): PET/CT at disease progression before treatment intensification (May 2026), showing multiple hypermetabolic liver metastases and local recurrence (SUVmax 18.2). (**D**): PET/CT obtained one month after multimodal treatment demonstrating a marked reduction in FDG uptake (SUVmax 6.6). Formal PERCIST assessment was not possible because SULpeak and standardized liver reference measurements were unavailable.

In February 2026, the patient developed local recurrence along with diffuse liver metastases; at this time his weight was 66 kg. The lesions partially regressed after local and systemic therapy, but in May 2026, he experienced clinical deterioration and marked tumor progression, with CA 19-9 increasing to 32.4 U/mL and his weight dropping to 58 kg. Pretreatment 18F-FDG PET/CT (May 2026) showed numerous, intensely FDG-avid liver metastases and increased metabolic activity at the site of local recurrence, with a peak SUVmax of 18.2 (Figure 1B). The dominant lesions were located in the pancreatic bed and liver.

At this point, the patient had nausea, vomiting, abdominal pain, right upper-quadrant and back pain, anorexia, weight loss, and a decline in performance status to ECOG PS 2.

Between February and May 2026, the patient continued systemic therapy while being evaluated for treatment modification because of progressive disease and increasing metabolic activity on PET/CT. The intensified multimodal regimen initiated in May 2026 is described in Section 2.4 and summarized in Table 1.

### 2.4. Therapeutic Intervention (May 2026)

The patient initially received a gemcitabine-based chemotherapy regimen in July 2023, consisting of gemcitabine, oxaliplatin, and 5-fluorouracil administered with 10–30% dose reductions. Following radiological disease progression in January 2025, treatment was changed to a second gemcitabine-containing combination regimen comprising paclitaxel, carboplatin, irinotecan, and gemcitabine, with dose reductions ranging from 20% to 50%. After further disease progression, accompanied by increased metabolic activity on PET/CT in May 2026, the chemotherapy regimen was intensified within the same multimodal treatment framework. Details of the systemic treatment regimens administered throughout the patient’s clinical course are summarized in Table 1.

In response to the increased metabolic activity observed on PET/CT, the chemotherapy component was modified into what we term a low-dose “activated” approach. In this report, “activated” chemotherapy refers to dose-reduced, frequently administered (metronomic-style) cytotoxic agents delivered under metabolically optimized conditions, including dietary carbohydrate restriction, regional hyperthermia, and hyperbaric oxygen therapy. The rationale was to enhance tumor perfusion, oxygenation, drug delivery, and chemosensitivity while minimizing treatment-related toxicity. A combination of methotrexate, vinorelbine, docetaxel, oxaliplatin, irinotecan, and gemcitabine was administered, with each cytotoxic agent given at no more than 50% of its conventional dose. Lenvatinib and everolimus were added at full doses on alternating days for two months. Regional hyperthermia and hyperbaric oxygen therapy were continued concurrently, together with dietitian-supervised carbohydrate restriction and metabolically focused supportive care.

Throughout all treatment phases, adjunctive therapies were administered concurrently. Hyperbaric oxygen therapy was delivered using a Quamvis 320 hyperbaric chamber (OxyHealth, LLC, Santa Fe Springs, CA, USA) at 1.5 atmospheres absolute (ATA) for 60–90 min per session on three consecutive days during each chemotherapy cycle. Regional hyperthermia was administered using an Oncotherm EHY-1030 system (Oncotherm GmbH, Troisdorf, Germany) for 60 min on three consecutive days, concurrent with chemotherapy. The patient also received a dietitian-supervised ketogenic diet with complete carbohydrate restriction and individualized caloric intake based primarily on protein- and fat-rich foods. Blood ketone concentrations were not routinely measured; therefore, nutritional ketosis could not be objectively confirmed.

The combined regimen was well tolerated. According to CTCAE v5.0, treatment-related adverse events included Grade 1 nausea, Grade 1 vomiting, and thrombocytopenia. No Grade 2 or higher non-hematological toxicity was observed. These events were manageable and did not require dose reduction, interruption, or discontinuation. The anorexia and weight loss noted at progression reflected disease activity rather than treatment toxicity and resolved after the response.

### 2.5. Follow-Up and Outcomes (June 2026)

About a month after the modified treatment began, the patient improved markedly. His nausea, vomiting, abdominal pain, and right upper-quadrant and back pain subsided, his appetite returned, and he began to regain weight (65 kg). Performance status recovered to ECOG PS 0.

Follow-up ^18^F-FDG PET/CT in June 2026 showed a pronounced decrease in FDG uptake across all regions compared with the previous study, with metabolic activity disappearing in many lesions (Figure 1C). In the dominant lesions, SUVmax decreased from 18.2 in May 2026 to 6.6 in June 2026—a 63.7% reduction in the target lesion—achieved after only one month of modified treatment. In parallel, CA 19-9 remained relatively stable, changing from 32.4 U/mL (May 2026) to 31 U/ML (June 2026).

Serial laboratory parameters measured throughout the patient’s clinical course are presented in Table 2.

### 2.6. Timeline

**Figure 2:**
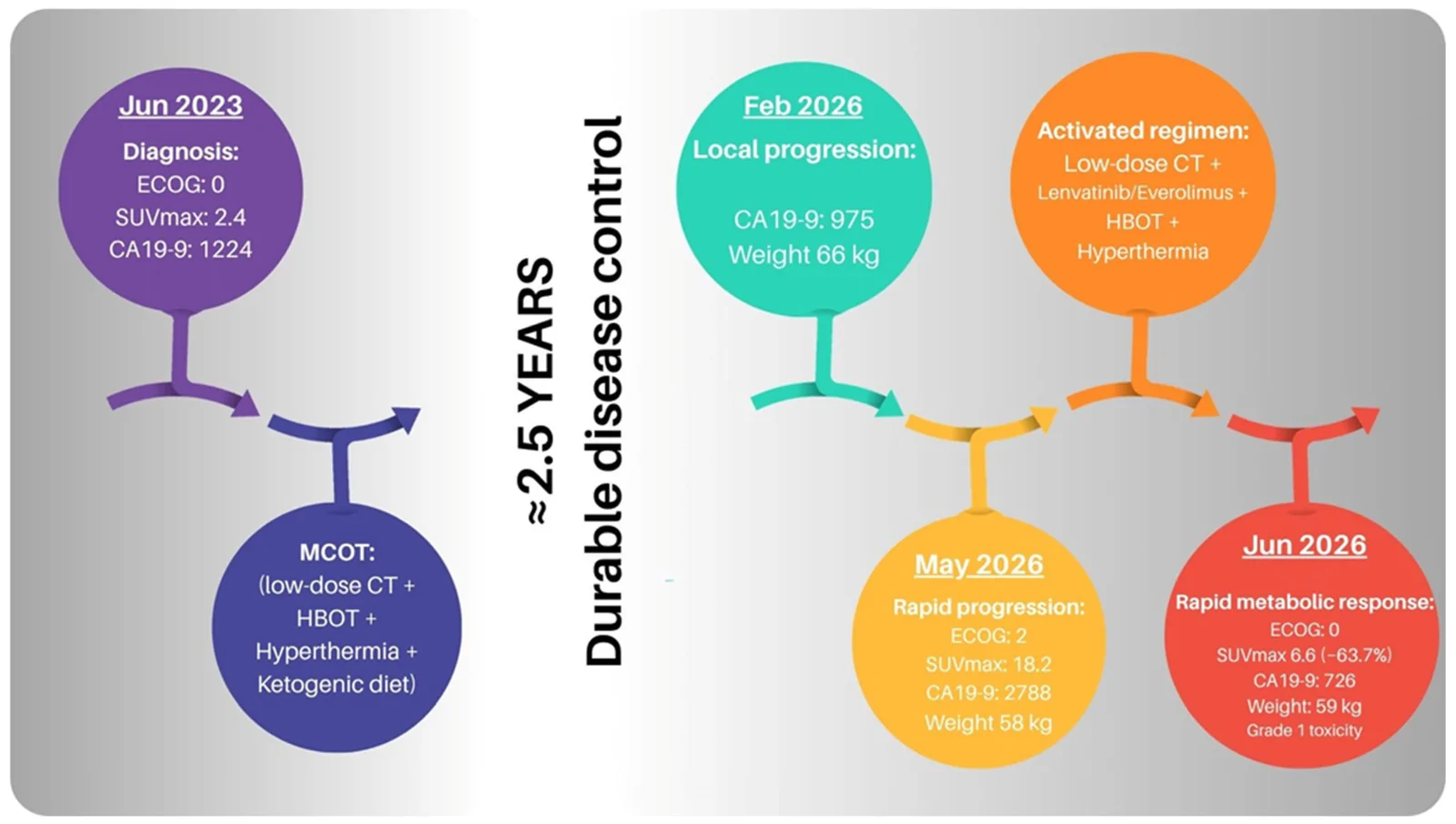
Clinical timeline and metabolic response following activated multimodal therapy. LA, locally advanced; PDAC, pancreatic ductal adenocarcinoma; MCOT, metabolically controlled oncologic therapy; CT, chemotherapy; HBOT, hyperbaric oxygen therapy.

The complete clinical course, including major clinical events, therapeutic interventions, and biomarker changes, is summarized as a timeline in Figure 2.

## 3. Discussion

This case documents a marked clinical and metabolic response that occurred soon after a simultaneous, multi-component change in therapy for refractory, advanced pancreatic adenocarcinoma. Hybrid molecular imaging with ^18^F-FDG PET/CT is an indispensable tool for quantifying tumor metabolic activity and assessing early therapeutic response in pancreatic adenocarcinoma. By integrating functional and anatomical information, PET/CT enables earlier evaluation of treatment efficacy than conventional morphological imaging alone and has become an important component of response assessment in oncology [12]. An early (approximately four-week) metabolic response has been shown to serve as an interim imaging biomarker for overall survival in pancreatic adenocarcinoma, while high FDG uptake (high SUVmax) is associated with worse survival [13]. The one-month reduction in SUVmax in our patient (18.2 to 6.6) may indicate an early metabolic response, although its prognostic significance cannot be established from a single case, and it was paralleled by a decrease in CA 19-9 over the same interval. Similarly, although the decline in CA 19-9 paralleled the metabolic response observed on PET/CT, CA 19-9 is influenced by several non-malignant conditions, including biliary obstruction and inflammation, and therefore should not be interpreted as a standalone predictor of treatment response.

### 3.1. Interpretation Within the PERCIST Framework and Its Limits Here

Metabolic response to therapy is ideally assessed with PERCIST 1.0, which standardizes 18F-FDG PET/CT response by using the peak lean-body-mass-normalized standardized uptake value (SULpeak) of a target lesion, measured within a 1 cm^3^ spherical volume of interest and referenced against background uptake in normal liver [14]. Under PERCIST, a partial metabolic response (PMR) requires at least a 30% decrease in SULpeak, and a complete metabolic response (CMR) is defined as the near-total disappearance of uptake in a measurable target lesion to the level of liver background [14].

Two methodological points require clarification. First, PERCIST is based on SULpeak, not SUVmax: SUL normalizes uptake to lean body mass, and SULpeak is the mean within a 1 cm^3^ peak volume rather than the single hottest voxel. Using the James predictive equation for a male patient, LBM = 1.10 × weight − 128 × (weight/height)^2^ (weight in kg, height in cm), the patient’s lean body mass was approximately 53.3 kg at recurrence (66 kg; February 2026), 48.9 kg at the pre-treatment scan (58 kg; May 2026), and 49.5 kg at follow-up (65 kg; June 2026); these values are needed to convert measured activity to SUL. Second, only SUVmax (18.2 → 6.6) was available retrospectively from the workstation slices; SULpeak and a measured liver reference (the mean SUL of a 3 cm spherical VOI in the right hepatic lobe, with its standard deviation) were not recorded. A formal PERCIST classification therefore cannot be assigned in this case.

With that caveat, the observed 63.7% decrease in tumor SUVmax far exceeds the 30% threshold that defines PMR, and the disappearance of activity in many lesions is qualitatively consistent with a pronounced partial—and, in some lesions, near-complete—metabolic response. This should be interpreted as supportive, indicative evidence rather than a validated PERCIST result. Prospective application of PERCIST in this setting would require recording SULpeak, the lean-body-mass normalization described above, and the liver reference threshold at each time point.

### 3.2. Comparison with Related Studies

The strategy used here aligns with reports of “metabolically supported chemotherapy” (MSCT). In 25 patients with metastatic pancreatic ductal adenocarcinoma, Iyikesici et al. combined a gemcitabine-based or FOLFIRINOX regimen (with induced hypoglycemia) with a ketogenic diet, hyperthermia, and hyperbaric oxygen, reporting a median overall survival of 15.8 months and a progression-free survival of 12.9 months [15]. An earlier series in unresectable (stage III–IV) disease reported a median survival of about 19.5 months [16]. Along with complete-response cases the same group has described in other tumor types, these reports provide practical support for the multimodal strategy used here.

Although most previous clinical reports of metabolically supported chemotherapy have originated from a single research group, independent preclinical and translational studies also support the biological rationale for combining metabolic interventions with systemic therapy in pancreatic cancer. Pancreatic tumors exhibit marked metabolic plasticity and dependence on altered nutrient utilization, making metabolic targeting an attractive therapeutic strategy [17]. Experimental studies have demonstrated synergistic effects between ketogenic diets and chemotherapy, and recent reviews have highlighted the potential importance of targeting tumor hypoxia and the metabolic microenvironment [18,19,20]. Carbohydrate restriction lowers the glucose–insulin tone on which glycolytic tumor cells depend. Recent preclinical studies have further shown that nutritional ketosis may act as a metabolic vehicle that enhances the efficacy of anticancer agents, permitting lower drug doses while maintaining therapeutic activity [21]. These observations support the concept of metabolic synergy, although clinical validation in pancreatic cancer is still lacking.

Low-dose, metronomic chemotherapy adds an independent rationale: continuous low-dose dosing can curb tumor growth through antiangiogenic effects, depletion of immunosuppressive cells, and support for dendritic cell maturation [22]. In pancreatic cancer models, low-dose gemcitabine reduced FDG uptake and increased apoptosis [10], supporting the plausibility of the dose-reduced, frequently administered regimen used in our patient.

### 3.3. Three-Year Survival: A Two-Stage Success

Perhaps the most striking feature of this case is that the response was not limited to a single month of metabolic regression but unfolded over approximately three years of overall survival, including approximately 2.5 years of durable disease control. In metastatic or locally advanced pancreatic adenocarcinoma, median overall survival with standard systemic therapy rarely exceeds 11–12 months [2,3]. Here, success appears in two distinct stages. In the first (2023–2026), the combined multimodal regimen (MCOT) achieved about two and a half years of continuous disease control—matching and even surpassing the median survival reported by Iyikesici et al. in their metabolically supported chemotherapy series (15.8 months; approximately 19.5 months in the unresectable cohort) [15,16]. In the second stage (May–June 2026), even as treatment-resistant progression emerged, the intensified low-dose regimen, administered under metabolically optimized conditions, produced a marked metabolic response within a month, with corresponding biochemical (CA 19-9) improvement and recovery of performance status from ECOG PS 2 to PS 0.

The three-time-point sequence in Figure 1 (February 2026 → May 2026 → June 2026) unifies this two-stage course into a single visual narrative: a prolonged period of disease control from diagnosis to progression, followed by metabolic suppression. Beyond the imaging and biochemical changes, the recovery of performance status from ECOG PS 2 to PS 0 within a month is arguably the most clinically meaningful outcome: the patient returned from a symptomatic, nearly bedridden state to full independence, regaining appetite and weight. In advanced pancreatic cancer, where treatment often imposes as much burden as the disease itself, such an improvement in functional status and quality of life—achieved with only Grade 1 toxicity—is a substantive endpoint in its own right, independent of survival. Taken together, the trajectory suggests this approach may offer not only an acute salvage response but also the potential for durable control. As noted below, however, no claim of causality or generalizable survival benefit can be made from a single case.

### 3.4. Novel Aspects

Several features distinguish this case. First, the response occurred in drug-resistant disease with diffuse liver metastases—a true salvage setting—whereas most MSCT series involve first-line or earlier-stage disease. Second, the speed: after just one month, regression was evident symptomatically, biochemically, and metabolically (SUVmax 18.2 → 6.6), supporting the feasibility of imaging-based early response assessment. Third, the regimen is explicitly multimodal, combining a dose-reduced multi-agent cytotoxic backbone (taxane, platinum, antifolate, antimetabolite, and topoisomerase inhibition) with two targeted agents (lenvatinib and everolimus) and physical modalities (hyperthermia and hyperbaric oxygen). This “multiple-vulnerability” design targets both tumor-cell bioenergetics and the microenvironment simultaneously. The metabolic and physical components, delivered alongside chemotherapy, distinguish this strategy from conventional dose reduction—and it was achieved with favorable tolerability.

Mechanistically, the rationale is based on the glycolytic phenotype of tumor cells (the Warburg effect) [7]: carbohydrate restriction lowers the glucose–insulin tone these cells depend on [8], hyperthermia improves perfusion and chemosensitivity, and hyperbaric oxygen counters hypoxia-driven resistance [9]. Together, these measures are intended to deliver chemotherapy under metabolically optimized conditions. However, this mechanistic rationale has not yet been confirmed by high-level clinical evidence, and these adjunctive modalities should currently be considered investigational. The rapid FDG response is, conceptually, consistent with this mechanism, though causality cannot be inferred from a single case.

### 3.5. Limitations

Because many distinct interventions were applied at once in a single patient (several cytotoxic agents, two targeted agents, hyperthermia, hyperbaric oxygen, and metabolic/microenvironmental support), the response cannot be ascribed to any one component, nor can the relative contribution of each be separated out. The evidence for hyperthermia, hyperbaric oxygen, and metabolic diets in pancreatic cancer is, moreover, limited and contested, and these measures do not replace standard treatment. In PET/CT images, only SUVmax was available, so SULpeak and the liver reference were not measured and the response could not be formally classified by PERCIST. No serial tissue biopsies or molecular characterization were available; therefore, the proposed metabolic activation is based on serial 18F-FDG PET/CT findings rather than direct biological evidence. In addition, spontaneous tumor fluctuation cannot be excluded, and the findings from this single uncontrolled observation should not be generalized to broader pancreatic cancer populations or used to guide clinical decision-making without prospective validation. Moreover, these metabolic adjuncts are not currently recommended in major international clinical guidelines for metastatic pancreatic adenocarcinoma and should be regarded as investigational adjunctive approaches rather than established standards of care. High-level prospective evidence demonstrating improvements in survival or treatment response is currently lacking. NGS was not performed in this patient; therefore, the potential contribution of underlying molecular alterations to the favorable clinical response remains unknown. Furthermore, the generalizability of this observation is inherently limited by patient selection. The patient was relatively young, had an ECOG performance status of 0 at diagnosis, and had no major comorbidities or adverse psychosocial factors that might have compromised treatment tolerance. Whether similar efficacy and tolerability could be achieved in older, frail, heavily pretreated patients or in patients with different molecular tumor profiles remains unknown. Further limitations include reliance on a single case, the absence of a randomized comparison and short-term follow-up of recurrence response duration at the time of publication. The findings are therefore hypothesis-generating rather than conclusive and must be confirmed in prospective, controlled studies.

## 4. Patient Perspective

The patient reported that, after starting the modified treatment, his nausea, abdominal and back pain, and appetite improved substantially within weeks, allowing him to eat, regain weight, and resume daily activities. He described, “After the new treatment, I was able to eat again, my pain decreased, and I tolerated the treatment well and wished to continue”.

## 5. Conclusions

In selected patients with advanced pancreatic cancer, a combined multimodal regimen of dose-reduced chemotherapy with metabolic and microenvironmental support may be associated with long-term disease control, as seen in this patient, whose approximately 2.5-year survival from diagnosis exceeded the expected median for metastatic disease. Even in heavily pretreated, drug-resistant patients with few remaining options, such a regimen may suppress tumor activity and symptoms with favorable tolerability. Because this observation is based on a single case, it cannot establish causality, and the findings should be tested in prospective, controlled studies.

## Figures and Tables

**Table 1:** Therapeutic interventions during the clinical course.

Phase	Period	Systemic Treatment	Dose Modification	Schedule
**Initial treatment**	July 2023	Gemcitabine, Oxaliplatin, 5-Fluorouracil	10–30% dose reduction	Days 1, 8, and 21
**First progression**	January 2025	Paclitaxel, Carboplatin, Irinotecan, Gemcitabine	20–50% dose reduction	Every 14 days
**Intensified treatment**	May 2026	Gemcitabine, Docetaxel, Oxaliplatin, Irinotecan, Vinorelbine	30–50% dose reduction	Every 14 days
**Targeted therapy**	May–July 2026	Lenvatinib 10 mg and Everolimus 10 mg	Full dose	Alternating daily for 2 months

Chemotherapy doses were individualized according to patient tolerance.

**Table 2:** Serial laboratory findings during the patient’s clinical course.

Parameter	Reference Range	10 Jul 2023 (Diagnosis)	14 Jan 2025	07 May 2026 (Before Modified Treatment)	17 Jun 2026 (After Treatment)
**Fasting blood glucose (mg/dL)**	70–99	131	117	121	85
**ALT (U/L)**	0–41	25	33	115	34
**AST (U/L)**	0–40	27	27	37	26
**ALP (U/L)**	40–129	72	86	360	295
**Urea (mg/dL)**	10–50	13	17.4	29	25
**Creatinine (mg/dL)**	0.7–1.3	0.90	0.74	0.36	0.67
**GGT (U/L)**	8–61	23	32	1110	706
**LDH (U/L)**	135–225	280	267	332	234
**WBC (×10^3^/µL)**	4.0–10.0	5.67	5.80	3.50	7.55
**Hemoglobin (g/dL)**	13.5–17.5	14.7	14.7	12.7	10.9
**Hematocrit (%)**	41–53	39.8	42.9	37.5	32.9
**Platelet (×10^3^/µL)**	150–400	83	117	284	57
**CEA (ng/mL)**	<5	6.2	ND	424	490
**CA 19-9 (U/mL)**	<37	6.2	ND	32.4	31

ALT, alanine aminotransferase; AST, aspartate aminotransferase; ALP, alkaline phosphatase; GGT, gamma-glutamyl transferase; LDH, lactate dehydrogenase; WBC, white blood cell count; CEA, carcinoembryonic antigen; CA 19-9, carbohydrate antigen 19-9; ND, not determined.

## Data Availability

The original contributions presented in this study are included in the article. Further inquiries can be directed to the corresponding author.
